# Evaluation of the World Health Organization-HEARTS hypertension control package in Bangladesh: a quasi-experimental trial

**DOI:** 10.1136/heartjnl-2024-324253

**Published:** 2024-07-16

**Authors:** Ahmad Abrar, Xiao Hu, Jubaida Akhtar, Shamim Jubayer, Mohammad Noor Nabi Sayem, Sarmin Sultana, Mohammad Abdullah Al Mamun, Mahfuzur Rahman Bhuiyan, Fazila Malik, Mohammad Robed Amin, Abdul Alim, Reena Gupta, Di Zhao, Margaret Farrell, Bolanle Banigbe, Kunihiro Matsushita, Daniel Burka, Lawrence Appel, Andrew E Moran, Sohel Reza Choudhury

**Affiliations:** 1 National Heart Foundation Hospital and Research Institute, Dhaka, Bangladesh; 2 Bloomberg School of Public Health, Johns Hopkins University, Baltimore, Maryland, USA; 3 Epidemiology, National Heart Foundation Hospital and Research Institute, Dhaka, Bangladesh; 4 Cardiology, National Heart Foundation Hospital and Research Institute, Dhaka, Bangladesh; 5 Directorate General of Health Services, Dhaka, Bangladesh; 6 General Medicine, University of California San Francisco, San Francisco, California, USA; 7 Resolve to Saves Lives, New York, New York, USA; 8 Johns Hopkins University, Baltimore, Maryland, USA; 9 Division of General Medicine, Columbia University Medical Center, New York, New York, USA

**Keywords:** hypertension, global health

## Abstract

**Background:**

The World Health Organization (WHO) promotes the HEARTS technical package for improving hypertension control worldwide, but its effectiveness has not been rigorously evaluated.

**Objective:**

To compare hypertension outcomes in clinics implementing HEARTS versus clinics continuing usual hypertension care in rural Bangladesh.

**Methods:**

A matched-pair cluster quasi-experimental trial in Upazila Health Complexes (UHCs; primary healthcare facilities) was conducted in rural Bangladesh. A total of 3935 patients (mean age 52.3 years, 70.5% female) with uncontrolled hypertension (blood pressure (BP) ≥140/90 mm Hg regardless of treatment history) were enrolled: 1950 patients from 7 HEARTS UHCs and 1985 patients from 7 matched usual care UHCs. The primary outcome was systolic BP at 6 months measured at the patient’s home; secondary outcomes were diastolic BP, hypertension control rate (<140/90 mm Hg) and loss to follow-up. Multivariable mixed-effects linear and Poisson models were conducted.

**Results:**

Baseline mean systolic BP was 158.4 mm Hg in the intervention group and 158.8 mm Hg in the usual care group. At 6 months, 95.5% of participants completed follow-up. Compared with usual care, the intervention significantly lowered systolic BP (−23.7 mm Hg vs −20.0 mm Hg; net difference −3.7 mm Hg (95% CI −5.1 to –2.2)) and diastolic BP (−10.2 mm Hg vs −8.3 mm Hg; net difference −1.9 mm Hg (95% CI −2.7 to –1.1)) and improved hypertension control (62.0% vs 49.7%, net difference 12.3% (95% CI 9.0 to 16.8)). Rate of missed clinic visits was lower in the intervention group (8.8% vs 39.3%, p<0.001).

**Conclusions:**

After WHO-HEARTS package implementation in rural Bangladesh, BP was lowered and hypertension control improved significantly compared with usual care.

**Trial registration number:**

NCT04992039.

**WHAT IS ALREADY KNOWN ON THIS TOPIC**

- It is well established that hypertension diagnosis, treatment and controlled blood pressure lower risks of cardiovascular disease events and deaths.
- The Global Hearts Initiative is implementing a standard WHO-HEARTS package for hypertension control in primary care clinics of 38 low- and middle-income countries.

**WHAT THIS STUDY ADDS**

- This quasi-experimental trial was completed alongside HEARTS programme expansion in rural Bangladesh and is the first to rigorously assess the complete HEARTS package for hypertension.

**HOW THIS STUDY MIGHT AFFECT RESEARCH, PRACTICE OR POLICY**

- WHO-HEARTS package implementation was feasible and effectively improved hypertension control in rural Bangladesh.
- The trial demonstrated that WHO-HEARTS is a standard and effective approach to improving hypertension control in low- and middle-income countries.

## Introduction

To improve hypertension control worldwide, World Health Organization (WHO) in 2016 developed the HEARTS technical package,^1^ a practical, public health approach to scaling up national hypertension control programmes that is consistent with WHO hypertension treatment guidelines.^2^ A total of 38 countries have implemented HEARTS, with 22.9 million patients treated worldwide by 2024.^3 4^ Implementation of HEARTS technical package-based hypertension control programmes has been described for programmes in Nigeria, Jordan, Chile, Cuba and other Caribbean countries,^5–8^ but the complete HEARTS package has never before been rigorously evaluated compared with a usual care control.

Of 21 million adults with hypertension in Bangladesh, 54% are unaware of their hypertension, 18% are diagnosed but untreated, 17% are treated but uncontrolled and only 11% have blood pressure (BP) controlled <140/90 mm Hg.^9^ To increase hypertension detection and control in Bangladesh, the Ministry of Health and Family Welfare and the National Heart Foundation of Bangladesh in collaboration with Resolve to Save Lives developed and launched the Bangladesh Hypertension Control Initiative (BHCI), a HEARTS hypertension control programme in four healthcare facilities in 2018. In the context of a 2020 expansion of the HEARTS programme to new healthcare facilities in rural Bangladesh, this study tested the effects of WHO-HEARTS package implementation on hypertension outcomes.

## Methods

### Study design

The Bangladesh HEARTS trial, a matched-pair cluster quasi-experimental trial, was conducted in 14 primary healthcare facilities named Upazila Health Complexes (UHCs) in rural Bangladesh to assess the effectiveness of WHO-HEARTS. Seven UHCs, six from Jamalpur district and one geographically similar UHC in Habiganj district, were were selected as usual care (control) UHCs from among 50 UHCs planning HEARTS package implementation under BHCI expansion after the 6 months of this study. Geographically isolated UHCs were selected purposely to reduce risk of cross-facility contamination. Another seven UHCs, similar in population size and literacy rate to the usual care UHCs, were selected from the remaining of the list as intervention sites.

### Study participants

Inclusion criteria were age ≥18 years, hypertension diagnosis (known prior diagnosis or a new diagnosis) and baseline systolic BP ≥140 mm Hg or diastolic BP ≥90 mm Hg (ie, ‘uncontrolled hypertension’) regardless of whether the person was taking antihypertensive medication. Exclusion criteria were current pregnancy, current treatment of an acute illness, terminal illness, or unwillingness to provide informed consent.

### Recruitment

An opportunistic hypertension screening protocol was implemented in intervention and usual care sites. All patients aged ≥18 years who presented to the outpatient reception desk of the UHC for a non-emergency visit had BP measured with an ‘arm in’ automated oscillometric digital BP device (A&D TM 2657P). For patients with systolic BP ≥130 mm Hg or diastolic BP ≥80 mm Hg on first measurement, a second BP was measured by a nurse, using the same ‘arm in’ digital BP device used for the first measurement, after a 2 min interval. If the second BP was systolic ≥140 mm Hg or diastolic ≥90 mm Hg, the patient was referred to a medical officer on the same day for a third, confirmatory BP using a validated desktop oscillometric digital BP measurement device (Omron Model HBP-1120). A hypertension diagnosis was made if the third, confirmatory BP was systolic ≥140 mm Hg or diastolic ≥90 mm Hg and the BP obtained in this measurement was considered as the baseline enrolment BP. In some instances, due to staffing shortages, trained field research staff measured second and third BP in place of a UHC nurse and Medical Officer; this was occasionally required in both intervention and usual care UHCs. Patients diagnosed with HTN were provided information about the HEARTS trial, and for those agreeing to participate in the trial, informed consent was obtained.

### Intervention: hypertension management

**Figure 1:**
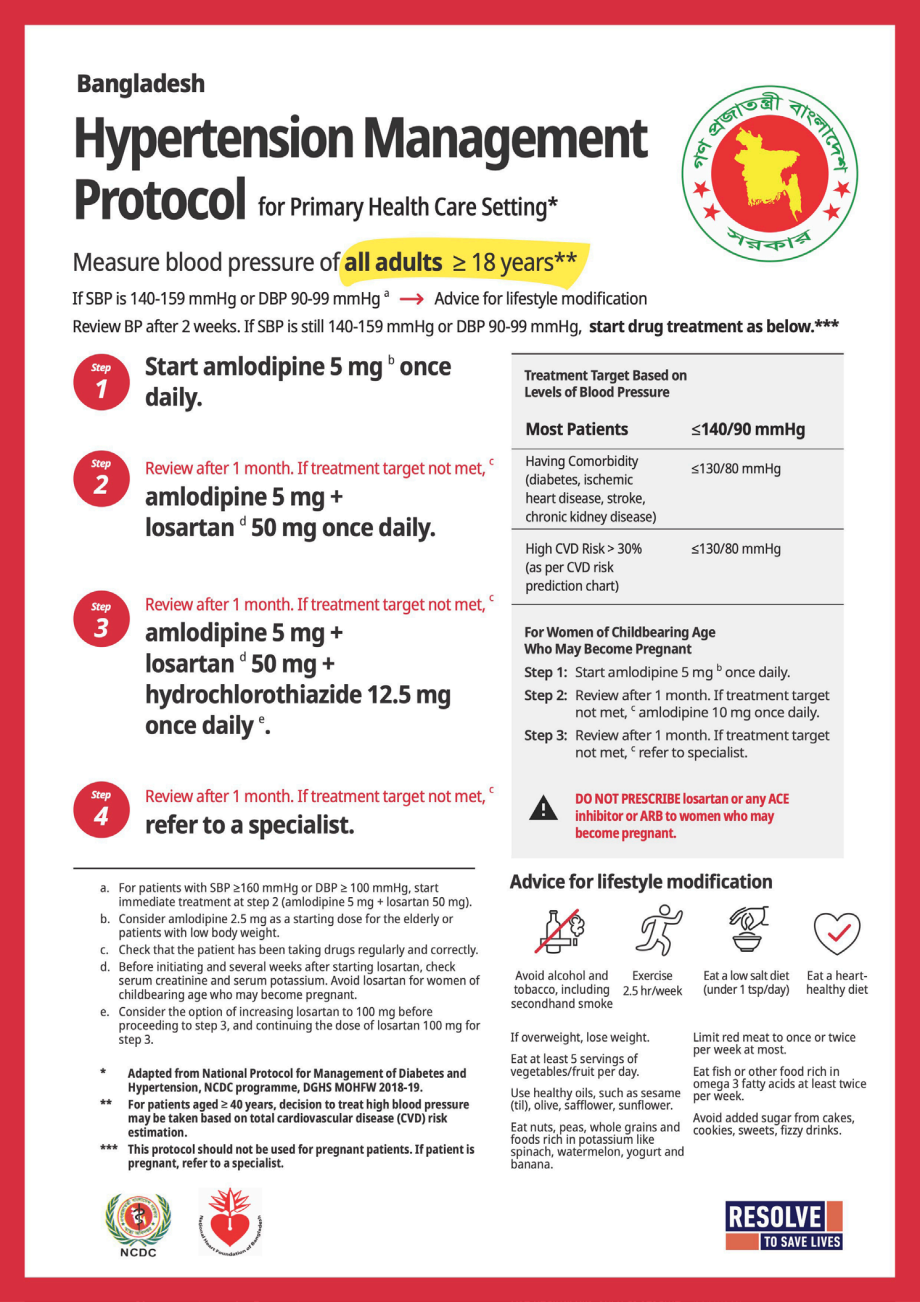
Drug-specific and dose-specific hypertension management protocol used in the Bangladesh HEARTS Hypertension Control Initiative. DBP, diastolic blood pressure; SBP, systolic blood pressure.

**Figure 2:**
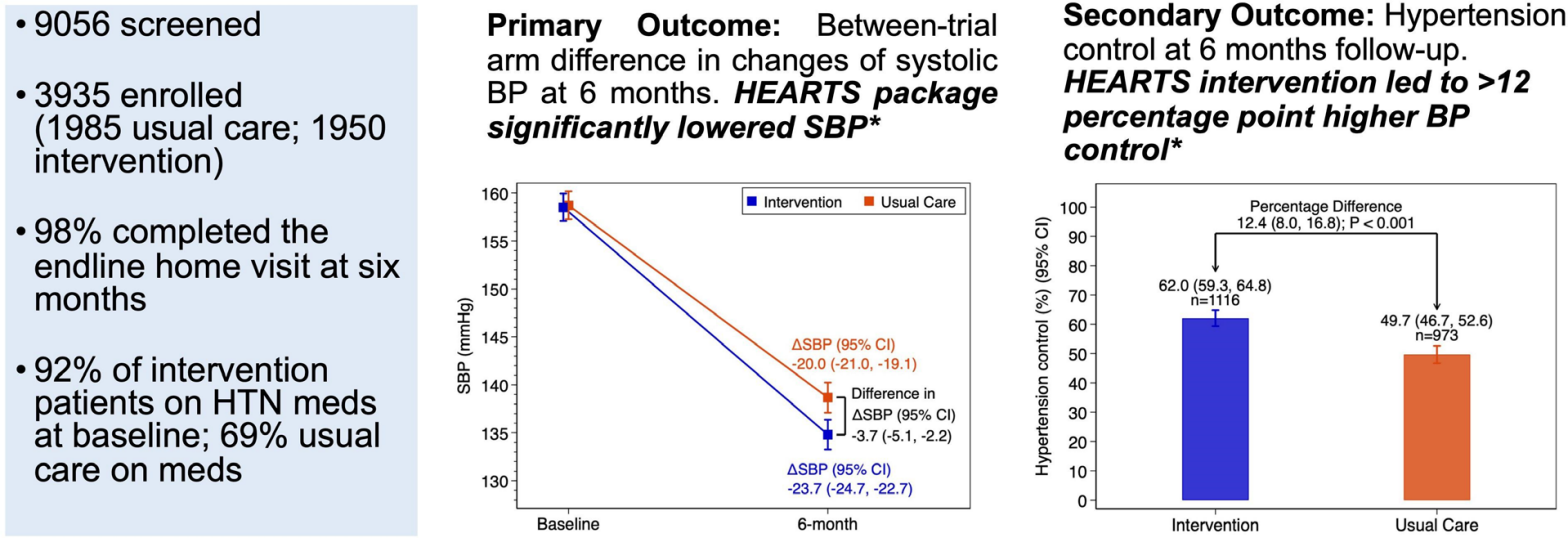
Central illustration: overview of the Bangladesh HEARTS trial. *Both models adjust for intervention, catchment area size, population, literacy rate, and other covariates (female vs male, age, diabetes, heart attack, stroke, CKD, prior use of antihypertensive medication, if flood prevents refill). Model for SBP additionally adjusted for interactions of visit with intervention and other covariates. BP, blood pressure; CKD, chronic kidney disease; HTN, hypertension; SBP, systolic blood pressure.

Consented patients with confirmed hypertension at intervention sites received hypertension care according to the WHO-HEARTS technical package. As shown in online supplemental table 1, key components of the HEARTS intervention were: (1) use of a three-step, drug-specific and dose-specific hypertension treatment protocol (figure 1), (2) standardised inventory and procurement practices to ensure 30-day prescriptions and a reliable supply of protocol medications in facility pharmacies, (3) team-based care involving medical officers (physicians), nurses and medical assistants, (4) a systematic approach to follow-up and retain patients in care and (5) a digital health information system (the Simple app) for registering patients into a secure database and tracking patient hypertension management and programme performance over time.^10^

10.1136/heartjnl-2024-324253.supp1 Supplementary data

Usual care site patients received the less structured hypertension management services commonly provided in UHCs before HEARTS implementation (online supplemental table 1). Specifically, there was no standardised treatment protocol, no expanded team-based care, no schedule of systematic patient follow-up and there were no added efforts to improve adherence and retention in care.

There were some features similar in both the intervention and usual care sites. Nurses were trained on BP measurement and to use the Simple app to record BP measurements and medication data. Both intervention and usual care site patients had access to hypertension medications free of charge procured and supplied by the government. Neither intervention nor usual care participants received any other financial incentive for participating in the study.

### Endline blood pressure outcomes

The primary outcome was difference between intervention and usual care sites in change of systolic BP from the baseline measurement to a follow-up measurement 6 months later. Because of severe flooding at several sites at approximately 6 months, follow-up was extended in both groups by 2 months, if needed, in order to complete follow-up visits to assess endline BP. Endline BP was measured in homes, instead of UHC clinics, to obtain high ascertainment of trial outcomes and minimise potential impact of differential loss-to-follow-up in the usual care and intervention sites. At the 6-month follow-up visit, research staff obtained triplicate measures of BP in the participants’ homes in intervention and usual care clusters using standard technique and the same Omron Model HBP-1120 used for baseline assessment. The average of the second and third readings represented each participant’s endline BP.

Secondary outcomes were between-group difference in change of diastolic BP, hypertension control rate at 6 months, clinic loss to follow-up and early follow-up rate. Hypertension control rate was defined as the number of enrolled patients with controlled BP (systolic BP <140 mm Hg and diastolic BP <90 mm Hg) measured in the community at 6 months divided by the number of enrolled patients who completed the 6-month follow-up visit. Clinic loss to follow-up rate was defined as the number of enrolled patients with no clinic visit through the entire 6-month follow-up period divided by the number of enrolled patients who completed the 6-month follow-up visit. Early follow-up rate was defined as enrolled patients with clinic visit within the first 3 months after enrolment divided by the number of enrolled patients who completed the 6-month follow-up visit.

### Endline questionnaire

At endline BP measurement visits, participants provided verbal responses to a standard questionnaire (online supplemental material appendix 1, online supplemental table 2). The questionnaire collected medication information including dose and dosage frequency, medicine names and doses, medication adherence for the last 7 days, experience of medicine-related adverse effects, incident hypertension-related complications or hospitalisations, impact of severe flooding on health services and other implementation outcomes.

### Statistical analysis

A sample size of 2100 was estimated to provide a minimal detectable difference of 5 mm Hg in systolic BP between intervention and usual care groups with 150 participants in each cluster, assuming a type I error of 5%, power of 80%, mean (SD) systolic BP of 148 (20) mm Hg at baseline, intracluster correlation coefficient of 0.01,^11^ follow-up rate of 50% (based on initial pilot experience) and a coefficient of variation of cluster size of 0.65.^12^

Descriptive statistics summarised baseline characteristics of the intervention and usual care sites. To evaluate the effect of intervention on the changes of systolic and diastolic BP over follow-up, we used linear mixed-effects models with a three-level hierarchical approach. The first level represents within-person variation with multiple BP measurements over time. The second level represents the variation of BP across participants, and the third level represents the variation across clusters. Between-group differences (95% CI) in BP change over 6 months were evaluated from the interaction term between the intervention group and the visit (follow-up vs baseline). Given the small number of clusters, we adopted unmatched analysis as the main statistical analytical method.^13^ Mixed-effects linear models were estimated via restricted maximum likelihood.

We also evaluated the effect of intervention on hypertension control rate, follow-up rate and patient-reported features of care, including the quality of hypertension care, reported improved BP management, planning to visit UHC again to receive ongoing treatment, spending less money coming to UHC compared with seeking treatment elsewhere and having billing problems. We used mixed-effects Poisson regression with robust variance to estimate the incidence rate ratio (IRR) and 95% CI for these outcomes comparing intervention versus usual care group. Additionally, we calculated the marginally adjusted rate of these outcomes for each group and the mean differences.

All analyses used a intention-to-treat analysis approach and adjusted for individual-level confounders including intervention group, age, sex, baseline assessment of history of heart attack, stroke, chronic kidney disease, diabetes, prior hypertension treatment, if severe flooding that occurred at about 6-month follow-up disrupted medication refill, plus cluster-level confounders including population size, area size and literacy rate (online supplemental table 3). For outcomes of BP change over time, we also incorporated interaction terms between scheduled routine clinic visit attendance and these baseline individual-level covariates. Participants missing the endline home visit were considered to have missing values on outcome measurements. Given the low percentage of missingness among all baseline participants (3.5% in the intervention arm, 5.5% in the usual care arm), we considered the impact of missing data to be minimal and did not conduct multiple imputation techniques to handle it.

Subgroup analyses were performed in strata defined by age, sex, baseline BP, hypertension medication use and diabetes. For sensitivity analyses, we used two-level mixed-effects models at the individual to estimate the differences in BP change within each matched cluster pair, then pooled the estimates from all cluster pairs together using meta-analysis random-effect model.

## Results

### Participant enrolment at baseline and follow-up at 6 months

**Figure 3:**
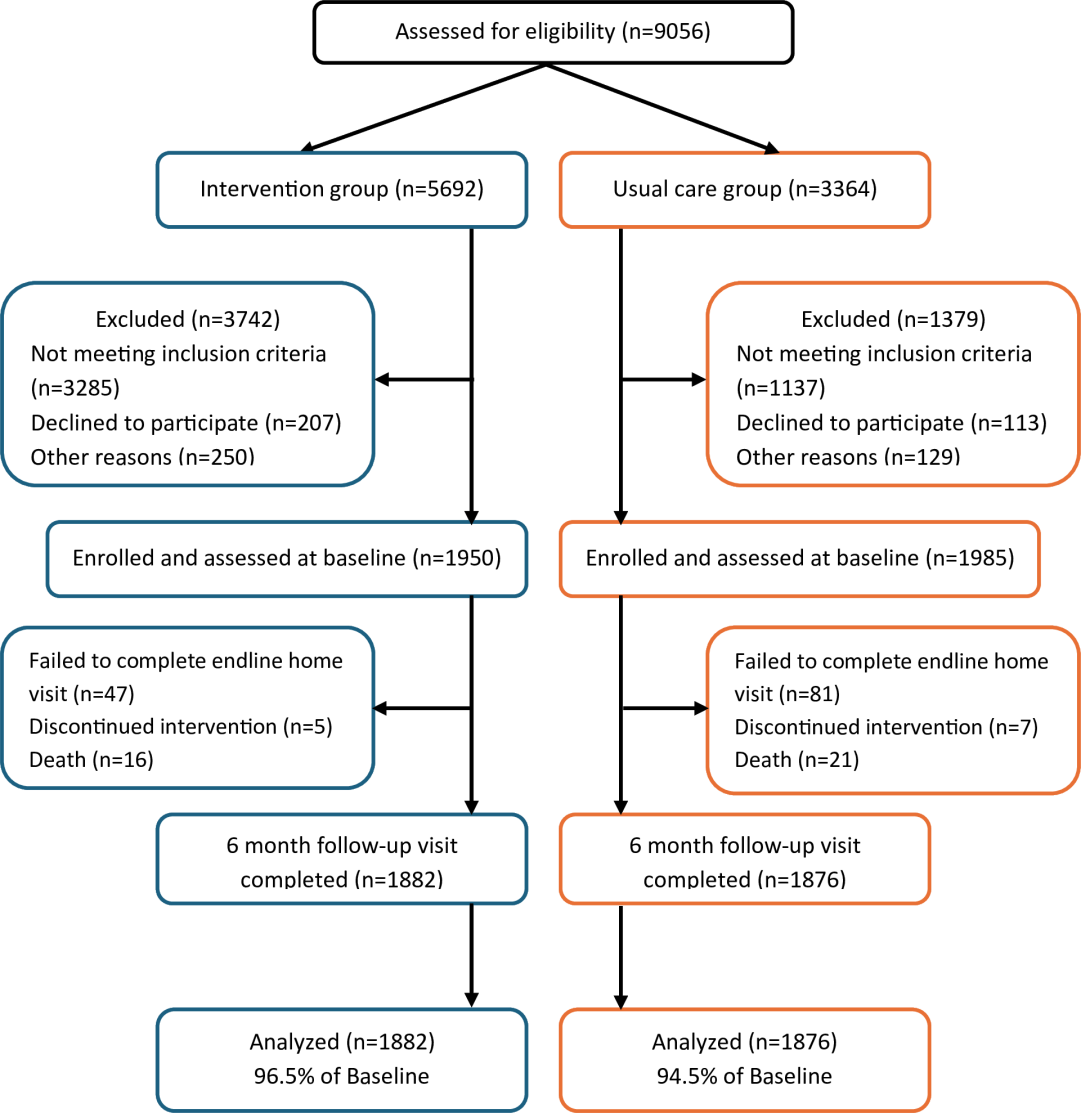
Inclusion and exclusion flow chart, the Bangladesh HEARTS trial.

Initially, there was a plan to enrol the calculated sample size of 2100 (1050 in each arm) over 3 months. Observing an overwhelming response from patients seeking hypertension care at intervention and usual care sites, researchers continued enrolment for 3 months as planned even after reaching target sample size. Among 9056 adults screened for eligibility (5692 in intervention group, 3364 in usual care), 5121 participants did not meet the inclusion criteria (3742 in intervention group, 1379 in usual care) (figure 2; figure 3). The study population therefore included 3935 participants (1950 in intervention group, 1985 in usual care group). The number of patients who failed to compete a home visit after 6 months was 47 (2.4% of enrolled) in intervention group and 81 (4.1% of enrolled) in usual care group. Twelve patients discontinued hypertension treatment at UHCs during follow-up (five in intervention and seven in usual care). Mean observation time (baseline to endline; ±SD) was 238.9±26.4 days in intervention group and 233.5±29.0 days in usual care group. There were 37 deaths during follow-up (16 in intervention; 21 in usual care). Finally, 3758 participants (95.5%) remained in the analysis (1882 intervention (96.5%); 1876 usual care group (94.5%)) after excluding those not available for endline visit follow-up at 6 months.

### Participant characteristics

At baseline, mean (±SD) age of the participants was 52.3±12.4 years, 70.5% were female; <1% had a history of heart attack, stroke or chronic kidney disease. Baseline characteristics were generally similar in the intervention and usual care; the intervention group had higher use of antihypertensive medication at baseline (92.2% vs 68.8%) and a higher diabetes prevalence (19.4% vs 7.5%; table 1, online supplemental table 4).

**Table 1:** Baseline characteristics, the Bangladesh HEARTS trial*

Characteristic	Total	Intervention	Usual care	Test	P value
Number	3935	1950	1985	Not applicable	
Female	2775 (70.5)	1350 (69.2)	1425 (71.8)	χ^2^	0.079
Age (years)	52.3 (12.4)	53.3 (12.2)	51.3 (12.5)	Two-sample t-test	<0.001
Diagnosis of diabetes	526 (13.4)	378 (19.4)	148 (7.5)	χ^2^	<0.001
Prior heart attack	20 (0.5)	10 (0.5)	10 (0.5)	Fisher’s exact	>0.99
Prior stroke	36 (0.9)	19 (1.0)	17 (0.9)	Fisher’s exact	0.74
Prior CKD	6 (0.2)	3 (0.2)	3 (0.2)	Fisher’s exact	>0.99
Baseline HTN medication use	3164 (80.4)	1798 (92.2)	1366 (68.8)	χ^2^	<0.001
Baseline SBP (mm Hg)	158.6 (15.2)	158.4 (15.0)	158.8 (15.4)	Two-sample t-test	0.48
Baseline DBP (mm Hg)	92.3 (10.3)	92.3 (10.2)	92.2 (10.3)	Two-sample t-test	0.86

Those with ‘unknown’ for prior disease history were assumed to have no relevant disease history.

*Data are presented as mean (SD) for continuous measures, and n (%) for categorical measures.

CKD, chronic kidney disease; DBP, diastolic blood pressure; HTN, hypertension; SBP, systolic blood pressure.

### BP change and hypertension control at 6 months

**Figure 4:**
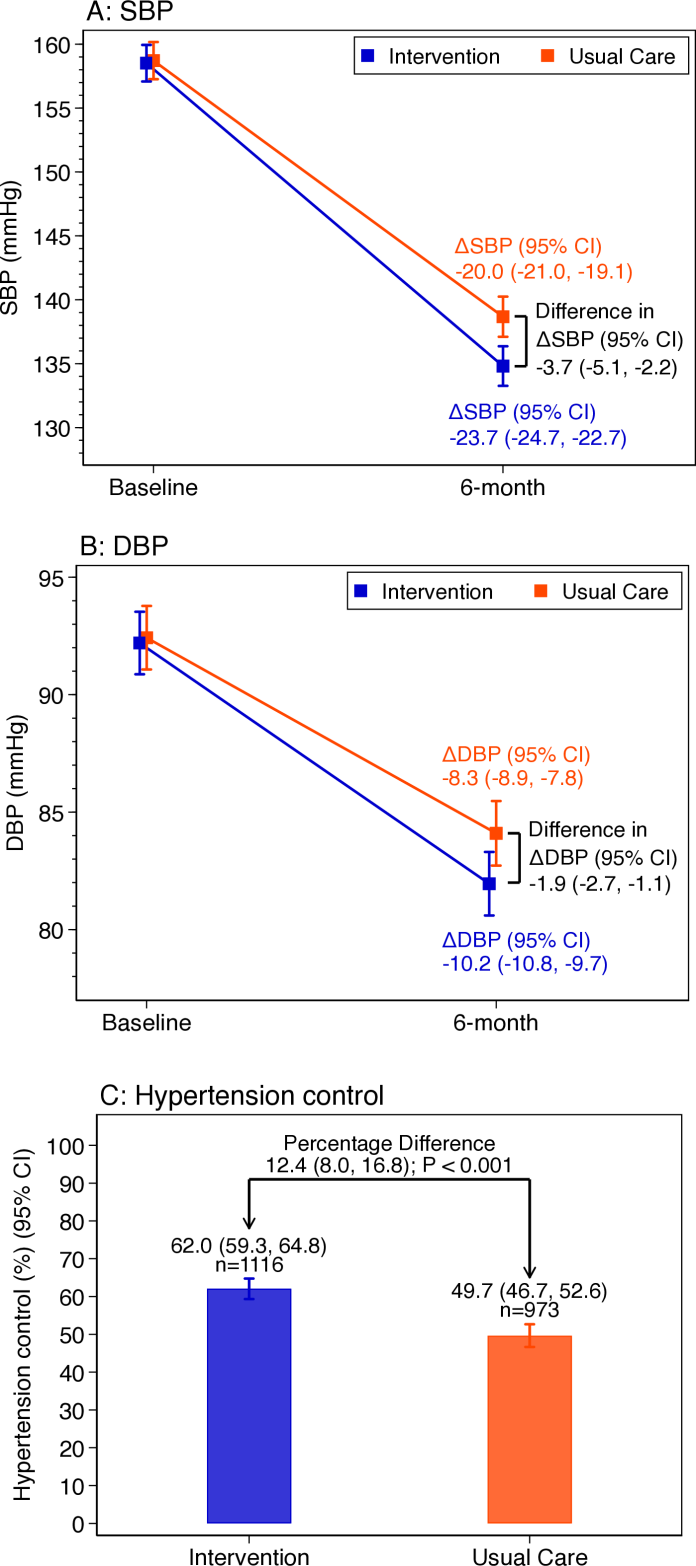
Adjusted between-group differences in hypertension control outcomes at the 6-month follow-up visit, the Bangladesh HEARTS trial. (A) Adjusted difference in mean baseline and endline systolic blood pressure. (B) Adjusted difference in mean baseline and endline diastolic blood pressure. (C) Adjusted difference in hypertension control. DBP, diastolic blood pressure; SBP, systolic blood pressure.

At 6-month follow-up, mean adjusted systolic BP was 23.7 (95% CI 22.7 to 24.7) mm Hg lower than baseline in intervention group and by 20.0 (95% CI 19.1 to 21.0) mm Hg lower than baseline in usual care. The intervention group experienced a greater systolic BP reduction than the usual care (mean adjusted difference −3.7 (95% CI −2.2 to −5.1) mm Hg; figure 4A). Mean adjusted diastolic BP was reduced by 10.2 (95% CI 9.7 to 10.8) mm Hg in intervention group and by 8.3 (95% CI 7.8 to 8.9) mm Hg in usual care. Intervention group experienced a greater diastolic BP reduction than the usual care (net adjusted difference of −1.9 (95% CI −1.1 to −2.7) mm Hg; figure 4B). At 6 months, hypertension control was 62.0% (95% CI 59.3% to 64.8%) in intervention group and 49.7% (95% CI 46.7 to 52.6%) in usual care (net adjusted difference of 12.4% (95% CI 8.0 to 16.8%; figure 4C). Results of crude analyses showed slightly lesser differences in BP and hypertension control (online supplemental figures 1A, 1B and 1C).

### Subgroup analyses

**Figure 5:**
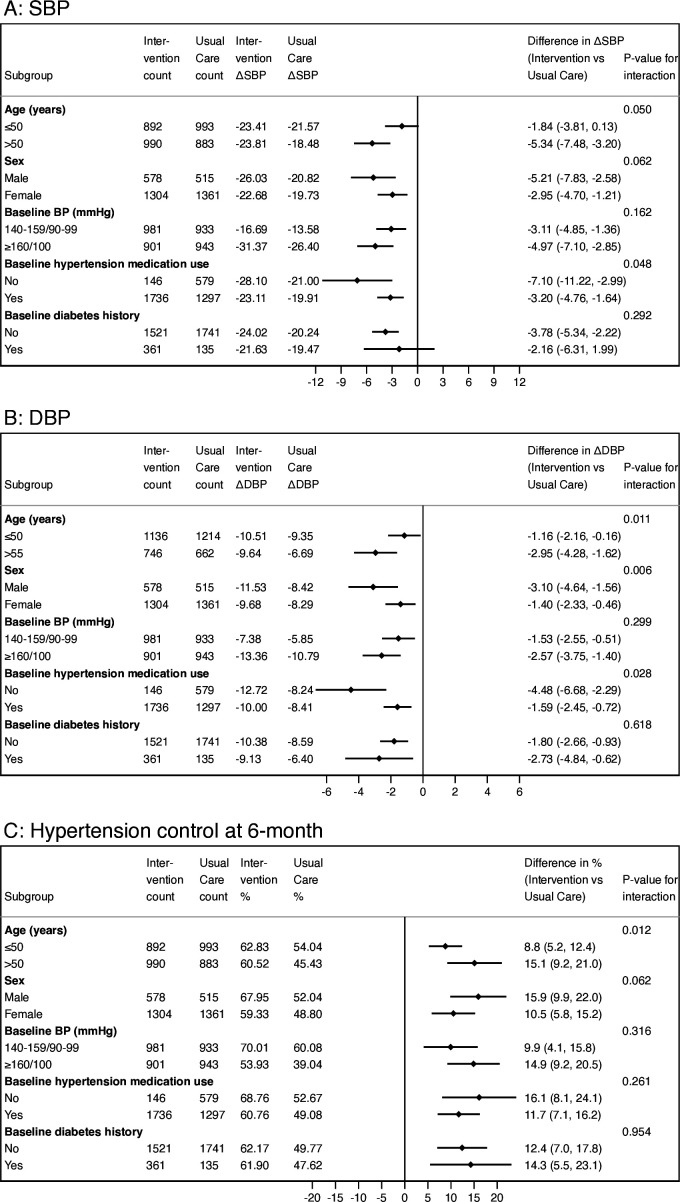
Subgroup analysis results for between-group differences in hypertension control outcomes at the 6-month follow-up visit, the Bangladesh HEARTS trial. (A) Change in systolic BP. (B) Change in diastolic BP. (C) Proportion with hypertension control. DBP, diastolic blood pressure; SBP, systolic blood pressure.

In prespecified subgroup analyses of systolic BP, diastolic BP and hypertension control (figure 5A–C), the effect of the intervention was always greater than that of the usual care; in several instances, the subgroup difference in between-group outcomes was statistically significant. The subgroups with greater benefits from the intervention group were persons aged ≥55 years, men and those not on medication at baseline.

### Implementation outcomes at 6 months

Compared with patients in the usual care group (table 2), patients in the HEARTS intervention group were less likely to miss scheduled clinic visits (IRR 0.22, 95% CI 0.15 to 0.32), and more likely to have a clinic visit <3 months after enrolment (IRR 1.67, 95% CI 1.49 to 1.87). Medication intensity score at endline, standardised for dose and frequency, was higher in the intervention group compared with the usual care patients (1.52, 95% CI 1.48 to 1.56 vs 1.01, 95% CI 0.97 to 1.05) (online supplemental tables 5–7).

**Table 2:** IRR for intervention and probability difference comparing intervention and control arm

	Clinic loss to follow-up*	Early follow-up†	Satisfied with HTN care‡	Improved HTN services§	Plan to visit again¶	Less money spent**	Problem paying medical bills††
IRR for intervention	**0.22 (0.15, 0.32**)	**1.67** (**1.49, 1.87**)	**1.13 (1.05, 1.22**)	**1.25 (1.05, 1.49**)	1.03 (0.99, 1.07)	**1.36** (**1.19, 1.56**)	1.00 (0.76, 1.32)
Count							
Intervention	150	1667	1731	1563	1773	1296	931
Control	802	919	1526	1188	1706	754	812
Marginal probability (%)							
Intervention	8.8 (6.1, 11.4)	85.3 (82.2, 88.3)	91.9 (90.3, 93.6)	81.2 (77.6, 84.8)	94.0 (92.8, 95.1)	88.6 (84.3, 92.8)	46.5 (41.9, 51.1)
Control	39.3 (33.9, 44.7)	50.9 (45.5, 56.3)	81.4 (75.3, 87.5)	64.8 (54.8, 74.8)	91.2 (87.8, 94.6)	65.0 (57.4, 72.5)	46.4 (34.2, 58.6)
Difference	**−30.5 (−37.3, –23.7**)	**34.3 (28.0, 40.6**)	**10.5 (4.3, 16.8**)	**16.4 (4.8, 28.1**)	2.8 (−0.8, 6.4)	**23.6 (14.3, 32.8**)	0.1 (−12.5, 12.8)

Mixed-effects Poisson model with robust variance was used. Model adjusted for intervention, female, age, diabetes, heart attack, stroke, CKD, prior HTN medication use, flood prevent refill, subdistrict area size, subdistrict population size, subdistrict literacy rate. Per cent and difference in per cent were calculated using marginal probability. Bold font indicates statistical significance (p<0.05).

*Defined as no clinic visit during the entire follow-up period.

†Defined as having clinic visit in the prior 3 months.

‡Satisfied with the quality of HTN care received at the UHC in the past 6 months.

§Improved HTN services, service received at the UHC in the past 6 months improved ability of HTN management.

¶Plan to visit UHC again to receive ongoing treatment.

**Spent less money since coming to UHC among those who used to seek treatment elsewhere (the comparison is among participants who received HTN treatment elsewhere before coming to UHC (n=2624)).

††Had times of being unable to pay medical bills in the past 12 months.

HTN, hypertension; IRR, incidence rate ratio; UHC, Upazila Health Complex.

Patients in the intervention group were more satisfied with the quality of hypertension care received at HEARTS UHCs (91.9% vs 81.4%, p=0.002), felt they had improved their ability to manage their hypertension (81.2% vs 64.8%, p=0.011) and spent less money for their hypertension care compared with usual care patients (88.6% vs 65.0%, p<0.001) (table 2). Patients in the intervention group had higher self-reported medication adherence and were more likely to be taking hypertension medications at follow-up (95.4% in the intervention group vs 75.7% in usual care group, p<0.001, and less likely to miss at least 1 day of medication in the last week 41.2% in intervention group and 99.9% in usual care group, p<0.001) (online supplemental table 8).

During the study, unexpected severe flooding impacted both intervention and usual care groups towards the end of follow-up. The intervention group was more affected by the flooding than the usual care group. Specifically, compared with patients in the usual care group, those in the intervention group reported the flood prevented them from attending ≥1 clinic visits (30% vs 8%, p<0.001) and prevented them from receiving at least one medication refill (27% vs 9%, p<0.001).

## Discussion

In this matched-pair cluster, quasi-experimental trial conducted in primary healthcare facilities in rural Bangladesh, we documented that WHO-HEARTS hypertension control package implementation significantly lowered both systolic and diastolic BP, and improved hypertension control compared with usual care. The HEARTS package appeared effective in all prespecified groups, with some evidence of greater benefits in older persons, men, those with newly diagnosed hypertension and those not taking antihypertensive medication at baseline.

The Bangladesh HEARTS trial align outcomes with other pragmatic trials of primary care-based hypertension control trials based in low- and middle-income countries. None of these studies specifically tested WHO-HEARTS, but they included some WHO-HEARTS components, for example, improved access to medicines, team-based, patient-centred approach and systematic follow-up.^14–17^ In these trials, conducted in Bangladesh/Pakistan/Sri Lanka, Argentina, Ghana and China, systolic BP reduction varied from 3.6 to 14.5 mm Hg greater in intervention compared with usual care. Between-group difference in hypertension control ranged from 5.2 to 32.5 percentage points. Notably, Bangladesh HEARTS observed outcomes only at 6 months, whereas the earlier trials observed outcomes between 12 and 24 months, and most did not observe substantial intervention benefits until after 6 months.

Limitations of the study include a quasi-experimental, rather than randomised trial design. Study design resulted in imbalances in some baseline characteristics, with more ‘hard-to-control’ patients (eg, with diabetes and uncontrolled BP despite medications) in the intervention group compared with the control group. Indeed, crude analysis associations were even strengthened after adjustment for multiple baseline covariates. Second, unexpected severe flooding interrupted clinic visits and medication refills and more in the intervention sites. It is possible that without the flooding, there would have been even greater benefits in the intervention group. Third, home measurements were selected because of concern that many patients would not have a clinic visit at 6 months and that there would be a substantial difference between usual care and intervention sites with the attendant risk of biased ascertainment. As table 2 shows, imbalance in clinic attendance did occur. In this context, the home BP, obtained in >95% of intervention and usual care participants, was a more unbiased assessment of trial outcomes (differences between groups). However, home-measured BP might overestimate magnitude of BP change from baseline and does not represent typical follow-up practice in Bangladesh. Nonetheless, clinic-measured and home-measured BP were implemented identically in intervention and usual care groups and thus between-group differences are valid. Fourth, the instruments for the patient-reported outcome measurements were developed by the researchers without validation, due to limited time and resources, which could limit confidence in the results.

In conclusion, in Bangladesh, the WHO-HEARTS package significantly lowered BP and improved hypertension control. These results provide evidence to scale-up the WHO-HEARTS hypertension control package in Bangladesh and support its implementation in other low- and middle-income countries.

## Data Availability

Data are available on reasonable request. De-identified data from this study are available on reasonable request.
