# Supplementary material for: Evaluation of the World Health Organization-HEARTS hypertension control package in Bangladesh: a quasi-experimental trial
Source: Heart. 2024 Jul 16;110(17):1090–8. doi: 10.1136/heartjnl-2024-324253 (PMC11347191; doi:10.1136/heartjnl-2024-324253)
Supplement: Supplementary data [file heartjnl-2024-324253supp001.pdf]

Supplemental Materials

Supplemental Methods

Questionnaire responses assessed between-group differences in implementation outcomes between the HEARTS intervention and usual care groups. In response to the questions related to level of satisfaction with the quality of hypertension care received at the UHC in the past six months, those who responded ‘satisfied’ or ‘very satisfied’ were categorized as satisfied. Responses indicating 'agree' or 'strongly agree' to the question on self-perceived improvement in hypertension self-management were classified as 'improved ability'. Responses from participants expressing ‘definitely yes’ or a ‘possibly’ about the intention to visit UHC again for continued hypertension treatment were classified as 'again'. Participants who received treatment for hypertension elsewhere before visiting the study UHC were asked to compare their experience about out-of-pocket expenditure for hypertension treatment. A response with ‘yes’ was considered as spending ‘less money’ since coming to UHC for hypertension treatment. Participants reporting they were unable to pay any medical bills in past 12 months were defined as having had a ‘bill problem’.

A medication intensity score was calculated by standardizing use of multiple antihypertensive drugs with different doses and dosage frequencies to assess the between-group difference in use of medication at endline. The endline questionnaire collected names, dosage and frequency of 17 antihypertensive medicines. To calculate the medication intensity score for one medicine, the prescribed daily dose for this medicine was set as the numerator. The denominator was the daily standard dose, as sourced from UpToDate (uptodate.com) and MayoClinic.org. For example, the standard dose for amlodipine is 5 mg (Supplementary Table 1). If a participant was taking amlodipine once a day with a 10 mg dosage each time, the score for amlodipine would be 10 mg*1/5 mg =2. If a medicine listed was not used by a participant, the score for this medicine was simply 0. This score was calculated for each individual medicine and summed up for all of the patient’s medicines to derive a total score, used as the medication

intensity score for that participant. The score was computed for each participant who completed the endline questionnaire. Adherence to the medication was assessed by self-reported missing one or more days of medication in the week prior to the endline follow-up visit.

Given the unexpected flooding, in the questionnaire, participants were also asked whether flooding prevented them from visiting the UHC or getting a refill of medication in the past 2 months.

Supplemental Tables

Supplemental Table 1: Components of the HEARTS technical package included or not included at intervention and usual care groups.

HEARTS component	Intervention	Usual care
Use of a drug-and dose-specific hypertension treatment protocol	X	-
Standardized inventory and procurement practices to ensure a reliable supply of protocol medications	X	-
Training and support for team-based model of hypertension care delivery	X	-
Standardized procedure for patient follow-up	X	-
Access to the Simple data dashboard for program monitoring by facility managers	X	-
Designated area for universal BP screening	X	X
A&D arm-in BP device for BP screening	X	X
Omron desktop BP device for confirmatory measurement by a medical officer	X	X
Site monitoring and mentorship to ensure universal screening and patient enrollment/registration	X	X
Training of UHC medical officers and nurses to measure BP using standard techniques	X	X
Training of NCD nurses (intervention sites)/UHC nurses (usual care sites) to pre-screen, obtain informed consent, enroll patients with hypertension, and conduct data collection via the Simple mobile smart-phone digital clinical hypertension information application (“app”)	X	X

Supplemental Table 2: Antihypertensive medicines included as treatment options in the endline survey, standard daily doses, and resulting medicine intensity scores. For participants taking more than one drug, the medicine intensity score for all of their individual drugs were summed.

Medication name	Dose option in survey	Standard dose (per day)	Score for dose option
**amlodipine**	5 mg	5 mg	1
	10 mg		2
**losartan**	25 mg	50 mg	0.5
	50 mg		1
	100 mg		2
**hydrochlorothiazide**	12.5 mg	12.5 mg	1
	25 mg		2
	50 mg		4
**bisoprolol**	2.5 mg	5 mg	0.5
	5 mg		1
**atenolol**	25 mg	50 mg	0.5
	50 mg		1
	100 mg		2
**olmesartan**	20 mg	20 mg	1
	40 mg		2
	80 mg		4
**telmisartan**	20 mg	40 mg	0.5
	40 mg		1
	80 mg		2
**propranolol**	20 mg	80 mg	0.25
	40 mg		0.5
**metoprolol**	25 mg	100 mg	0.25
	50 mg		0.5
	100 mg		1
**chlorthalidone**	12.5 mg	25 mg	0.5
	25 mg		1
**spironolactone**	12.5 mg	25 mg	0.5
	25 mg		1
	50 mg		2
**lisinopril**	5 mg	10 mg	0.5
	10 mg		1

	20 mg		2
	40 mg		4
**captopril**	12.5 mg	50 mg	0.25
	25 mg		0.5
	50 mg		1
**enalapril**	2.5 mg	5 mg	0.5
	5 mg		1
	10 mg		2
	20 mg		4
	40 mg		8
**carvedilol**	6.25 mg	12.5 mg	0.5
	12.5 mg		1
**nifedipine**	20 mg	20 mg	1
**prazosin**	0.5 mg	2 mg	0.25
	1 mg		0.5

Note: For each medicine, the frequency included two options: 1) once per day, and 2) twice per day. The intensity score for one medicine equals the score for the dose option multiplied by the frequency. For example, if one participant takes hydrochlorothiazide at 12.5 mg per dose and twice a day, then the score for the dose option is 1, and the intensity score for hydrochlorothiazide is 1×2=2.

Supplemental table 3: Model coefficient for covariates in final model

Variables	SBP		DBP		Hypertension under control at 6-month	
	β	95% CI	β	95% CI	IRR	95% CI
Intervention	-0.20	[-2.39, 1.98]	-0.22	[-2.26, 1.81]	1.25***	[1.15, 1.35]
Visit	-20.04***	[-21.03, -19.05]	-8.33***	[-8.87, -7.78]	-	-
Intervention*visit	-3.66***	[-5.11, -2.21]	-1.92***	[-2.72, -1.12]	-	-
Female	1.90***	[0.81, 2.99]	-1.67***	[-2.37, -0.97]	0.85***	[0.79, 0.92]
Age	0.24***	[0.20, 0.28]	-0.29***	[-0.31, -0.26]	0.99***	[0.99, 1.00]
Diabetes	-0.91	[-2.37, 0.54]	-2.32***	[-3.26, -1.39]	0.98	[0.92, 1.05]
Heart attack	0.94	[-6.18, 8.06]	0.21	[-4.36, 4.77]	1.19	[0.89, 1.60]
Stroke	-2.14	[-7.37, 3.09]	-2.13	[-5.48, 1.23]	1.09	[0.80, 1.48]
CKD	-7.22	[-19.40, 4.96]	1.04	[-6.77, 8.86]	1.08	[0.57, 2.06]
HTN medication	0.88	[-0.40, 2.15]	0.66	[-0.16, 1.48]	0.97	[0.89, 1.05]
Flood prevents refill	-	-	-	-	0.93	[0.85, 1.01]
Area size	-0.01	[-0.03, 0.00]	0.01	[-0.00, 0.03]	1.00***	[1.00, 1.00]
Population size	0.02	[-0.01, 0.04]	-0.02	[-0.04, 0.01]	1.00	[1.00, 1.00]
Literacy rate	-0.16	[-0.47, 0.16]	0.08	[-0.22, 0.38]	1.00	[0.99, 1.01]
Female*visit	2.74***	[1.21, 4.28]	1.79***	[0.95, 2.64]	-	-
Age*visit	0.09**	[0.03, 0.15]	0.11***	[0.08, 0.14]	-	-
Diabetes*visit	1.73	[-0.28, 3.75]	1.50**	[0.38, 2.61]	-	-
Heart attack*visit	-2.56	[-12.60, 7.48]	-2.58	[-8.11, 2.95]	-	-
Stroke*visit	2.06	[-5.32, 9.44]	-0.40	[-4.47, 3.66]	-	-
CKD*visit	3.60	[-13.61, 20.80]	-0.28	[-9.76, 9.20]	-	-
HTN	1.34	[-0.44, 3.12]	0.08	[-0.90, 1.06]	-	-

medication*visit						
Flood prevents refill*visit	3.91***	[2.28, 5.54]	0.74	[-0.14, 1.62]	-	-
Intercept	158.71***	[157.26, 160.17]	92.42***	[91.07, 93.78]	0.68**	[0.54, 0.86]

Supplemental Table 4: Baseline characteristics (among 3758 participants included in the analysis)

	**Total**	**Intervention**	**Usual Care**	**Test**	p-value
	N=3,758	N=1,882	N=1,876		

Female	2,665 (70.9)	1,304 (69.3)	1,361 (72.5)	Chi-square	0.028
Age (years)	52.2 (12.2)	53.2 (12.1)	51.3 (12.3)	Two sample t test	<0.001
Diagnosis of diabetes	496 (13.2)	361 (19.2)	135 (7.2)	Chi-square	<0.001
Prior heart attack	18 (0.5)	9 (0.5)	9 (0.5)	Fisher's exact	>0.99
Prior stroke	33 (0.9)	19 (1.0)	14 (0.7)	Fisher's exact	0.48
Prior CKD	6 (0.2)	3 (0.2)	3 (0.2)	Fisher's exact	>0.99
Baseline HTN medication use	3,033 (80.7)	1,736 (92.2)	1,297 (69.1)	Chi-square	<0.001
Baseline SBP (mmHg)	158.6 (15.2)	158.4 (15.0)	158.9 (15.4)	Two sample t test	0.30
Baseline DBP (mmHg)	92.3 (10.3)	92.3 (10.2)	92.3 (10.3)	Two sample t test	0.84

*Data are presented as mean (SD) for continuous measures, and n (%) for categorical measures.
Abbreviations: SBP- systolic blood pressure; DBP- diastolic blood pressure, HTN- hypertension, CKD-chronic kidney disease.
Those with “unknown” for prior disease history were assumed to have no relevant disease history.

Supplemental table 5: Mean number of medication and medication intensity score by arms

	Intervention	Usual care	p-value
	N=1,882	N=1,876	
Number of medication (mean [SD])			
At Baseline	1.38 (0.58)	1.22 (0.72)	
At endline	1.47 (0.79)	0.98 (0.73)	
Difference from baseline to endline	0.09 (0.79)	-0.24 (0.92)	<0.001
Medication intensity score at endline (mean [SD])			
At endline	1.52 (0.89)	1.01 (0.81)	<0.001

Note:

p-value were from two-sample t-test.

Baseline and endline medication information was collected in different ways. Endline medication was a series of “yes/no” question to 17 medicines. The total number of medications was the total number of “yes” answers. Baseline medication was free text recording medicine names and each participant report up to 5 medications. The free text data was cleaned and managed to count the number of medicines.

Supplemental table 6: Number of prescribed antihypertensive medications at baseline enrollment

	Total	Intervention	Usual care	p-value
	N=3,758	N=1,882	N=1,876	
Total number of prescribed antihypertensive medication at baseline				<0.001
0	245 (6.5)	34 (1.8)	211 (11.2)	
1	2,301 (61.2)	1,157 (61.5)	1,144 (61.0)	
2	1,073 (28.6)	639 (34.0)	434 (23.1)	
3	120 (3.2)	48 (2.6)	72 (3.8)	
4	18 (0.5)	4 (0.2)	14 (0.7)	
5	1 (0.0)	0 (0.0)	1 (0.1)	

Note:pP-value were from Chi-square test.

Supplemental table 7: Number of antihypertensive medications used at endline.

	Total	Intervention	Usual care	p-value
	N=3,758	N=1,882	N=1,876	
Total number of antihypertensive medications used at endline				<0.001
0	694 (18.5)	200 (10.6)	494 (26.3)	
1	1,702 (45.3)	747 (39.7)	955 (50.9)	
2	1,192 (31.7)	791 (42.0)	401 (21.4)	
3	167 (4.4)	141 (7.5)	26 (1.4)	
4	3 (0.1)	3 (0.2)	0 (0.0)	

Note: p-value were from Chi-square test.

Supplemental table 8: Medication adherence at 6-month endline follow up visit

Outcome	Intervention	Control	p-value
	N=1,882	N=1,876	
Taking prescribed medication at endline	1795 (95.4%)	1420 (75.7%)	<0.001
Missed at least one day of medication in the last week	775 (41.2%)	1875 (99.9%)	<0.001

Note: p-value were from Chi-square test.

Supplemental figure 1a: crude systolic blood pressure levels, change in systolic blood pressure at 6 months, and difference in systolic blood pressure change at 6 months

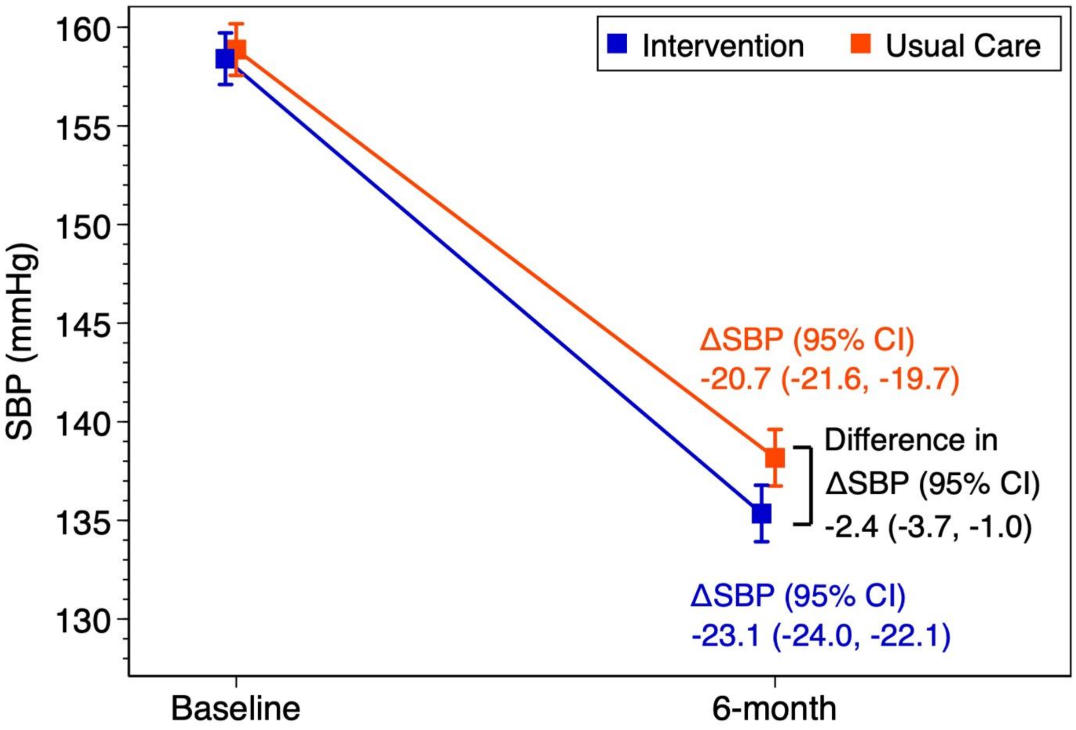

Supplemental figure 1b: crude diastolic blood pressure levels, change in diastolic blood pressure at 6 months, and difference in diastolic blood pressure change at 6 months

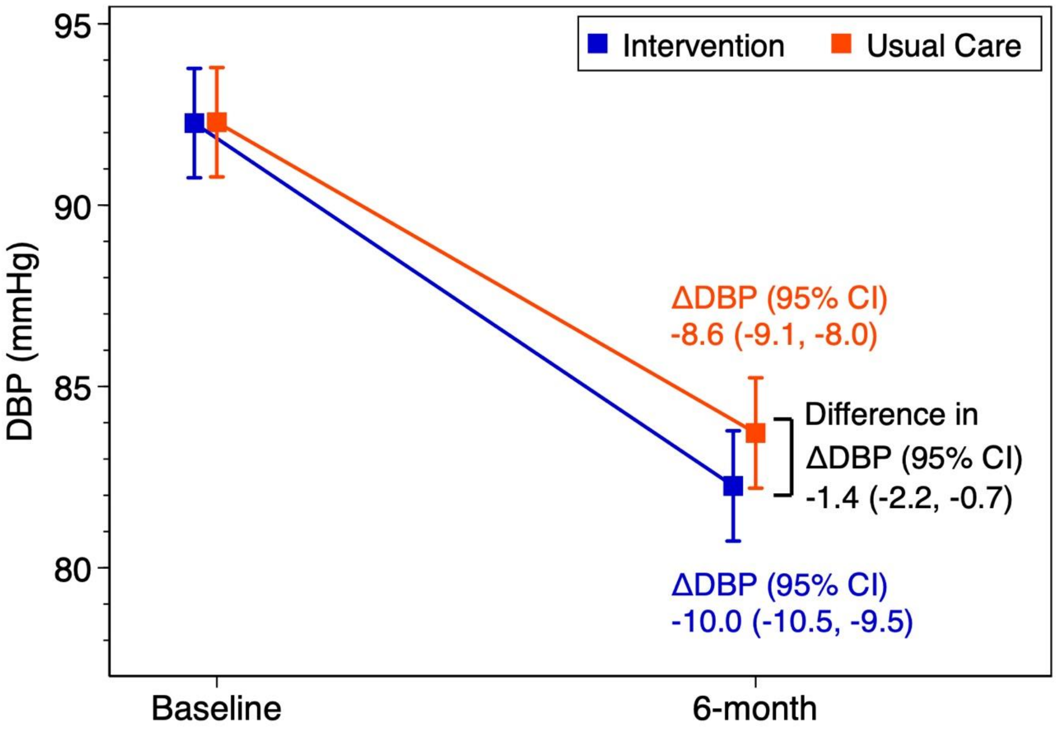

Supplemental figure 1c: Crude hypertension control at 6 months

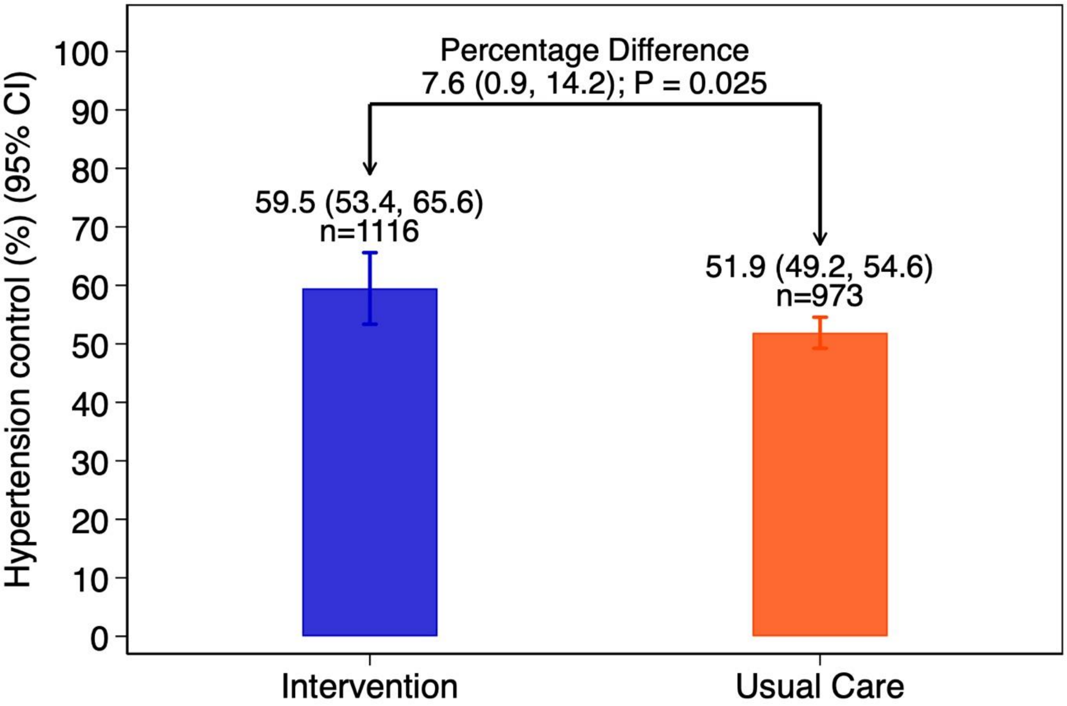

Supplemental figure 2: cluster level analysis for systolic BP and diastolic BP

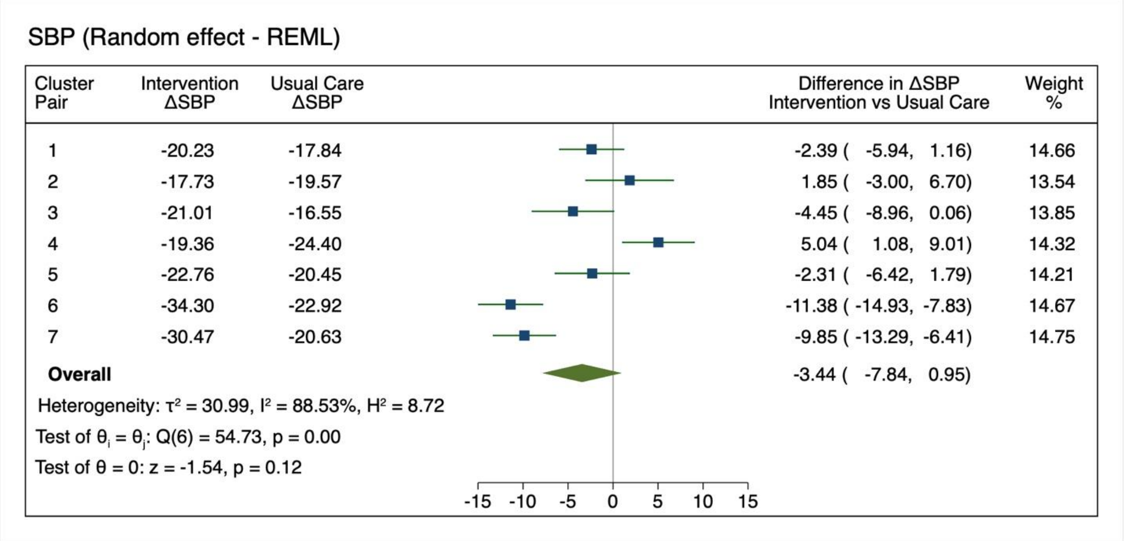

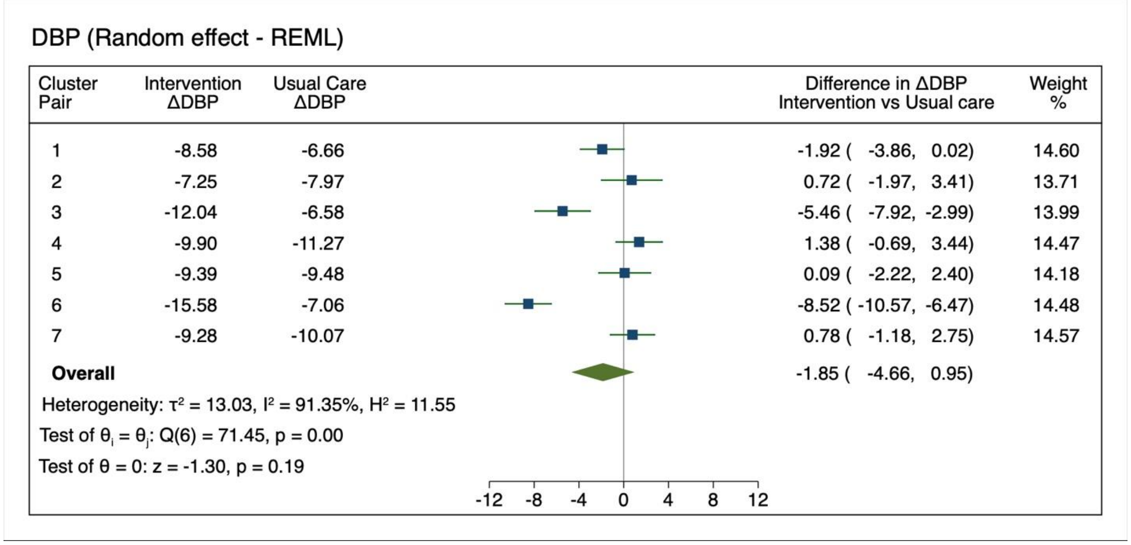

Note:

- **For each matched pair, linear mixed effect models** were used with random intercept at individual level and random slope for visit.
- **Model adjusted for** intervention, visit, intervention*visit, female, age, diabetes, heart attack, stroke, CKD, prior antihypertensive medication use, and interaction between visit and female, age, diabetes, heart attack, stroke, prior antihypertensive medication use, flood prevent refill.
- Estimates from all pairs were pooled together using meta-analysis random-effects model.

Supplemental Appendix 1: Endline questionnaire

1. Did you take your blood pressure medicine this morning/ last evening (i.e., last dose)?
   i. Yes
   ii. No

2. Over the past 7 days, how many days did you miss a dose of your blood pressure medicine?
   ______ days.

3. Which medication(s) are you currently taking for your high blood pressure? Please mention with dose and dosage frequency

Name (Generic)	Dose	Frequency
Amlodipine	5 mg<br>10 mg	Once in a day<br>twice in a day
Atenolol	25 mg<br>50 mg<br>100 mg	Once in a day<br>twice in a day
Captopril	12.5 mg<br>25 mg<br>50 mg	Once in a day<br>twice in a day
Chlorthalidone	12.5 mg<br>25 mg	Once in a day<br>twice in a day
Enalapril	2.5 mg<br>5 mg<br>10 mg<br>20 mg<br>40 mg	Once in a day<br>twice in a day
Hydrochlorothiazide	12.5 mg<br>25 mg<br>50 mg	Once in a day<br>twice in a day
Lisinopril	5 mg<br>10 mg<br>20 mg<br>40 mg	Once in a day<br>twice in a day
Losartan	25 mg<br>50 mg<br>100 mg	Once in a day<br>twice in a day
Metoprolol	25 mg<br>50 mg<br>100 mg	Once in a day<br>twice in a day

Propranolol	20 mg<br>40 mg	Once in a day<br>twice in a day
Spironolactone	12.5 mg<br>25 mg<br>50 mg	Once in a day<br>twice in a day
Telmisartan	20 mg<br>40 mg<br>80 mg	Once in a day<br>twice in a day
Bisoprolol	2.5 mg<br>5 mg	Once in a day<br>twice in a day
Carvedilol	6.25 mg<br>12.5 mg	Once in a day<br>twice in a day
Nifedipine	20 mg	Once in a day<br>twice in a day
Prazosin	0.5 mg<br>1 mg	Once in a day<br>twice in a day
Olmesartan	20 mg<br>40 mg	Once in a day<br>twice in a day
Other (Please specify)	…….. mg	Once in a day<br>twice in a day

4. Is any of these medicines combined into a single pill? (Write from observation)
   a. Yes
   b. No

If yes, proceed to 5, If no, skip to 6.

5. What are the combination drugs?

Amlodipine + Atenolol
Amlodipine + Olmesartan
Amlodipine + Valsartan
Amlodipine + Telmisartan
Amlodipine + Bisoprolol
Bisoprolol + Hydrochlorothiazide
Irbesartan + Hydrochlorothiazide
Losartan + Hydrochlorothiazide
Olmesartan + Hydrochlorothiazide
Valsartan + Hydrochlorothiazide
Telmisartan + Hydrochlorothiazide
Hydrochlorothiazide + Triamterene
Other (Please specify)

6. This is a list of problems people sometimes experience after taking blood pressure medication. Did you feel any of these symptoms during the past two weeks?

Symptoms	Response
Swelling of legs or ankles	i. Yes<br>ii. No
Dizziness/ lightheadedness	i. Yes<br>ii. No
Palpitations	i. Yes<br>ii. No
Stomach upset (Nausea, vomiting, abdominal pain)	i. Yes<br>ii. No
Cough	i. Yes<br>ii. No
Weakness	i. Yes<br>ii. No
Any other symptom (Please specify)	

7. Did you have any of these in the past 6-7 months since you were enrolled in the hypertension study? (Diagnosed by a doctor and documented)

Symptoms	Response
Stroke	i. Yes<br>ii. No
Heart attack	iii. Yes<br>iv. No
Kidney disease	iii. Yes<br>iv. No

8. Did you need to be get admitted into hospital in the past 6-7 months since you were enrolled in the hypertension study?
   a. Yes
   b. No

If yes, proceed to 9, If no, skip to 10.

9. Cause of hospital admission (Recorded from the hospital documents i.e., discharge certificate)

10. Did you seek treatment for HTN anywhere other than the UHC in the past 6-7 months since you were enrolled in the hypertension study?
    a. Yes
    b. No

11. If yes, where did you seek treatment?
    a. Community clinic
    b. Union health complex
    c. District hospital
    d. Medical college hospital
    e. Local pharmacy
    f. Private physician
    g. Private hospital
    h. Alternative medicine (Homeopathic/ Ayurvedic/Unani)
    i. Other (please specify)

12. The last 3 times you visited the UHC, did you receive free medications for hypertension?
    a. Yes, hypertension medications were available at every visit to the UHC
    b. No, hypertension medications were NOT available at every visit to the UHC

13. If no, how many times in the last three visits did you not receive free medications for HTN?
    a. 1 time
    b. 2 times
    c. 3 times

14. How satisfied are you with the quality of hypertension care you received at the UHC in the past 6-7 months?

Very unsatisfied	Unsatisfied	Neither Satisfied or Unsatisfied	Satisfied	Very satisfied
1	2	3	4	5

15. The services I received at the UHC in the past 6 months have improved my ability to effectively manage my hypertension

Strongly disagree	Disagree	Neither Agree or Disagree	Agree	Strongly agree
1	2	3	4	5

16. Do you plan to visit again to the UHC to receive ongoing treatment?

Definitely not	Unlikely	Neither yes or no	Possibly	Definitely yes
1	2	3	4	5

If yes, proceed to 17, If no, skip to 18.

17. If answer 1 or 2, why not? (select all that apply)

a. Distance to travel to UHC
b. Transportation costs
c. Time away from work/other duties/lost wages
d. Seeking care at private/other facility
e. Do not have confidence in services/medications received at UHC
f. Moved to other location
e. Other, please specify ____________________

18. Before you started coming to the UHC, were you receiving treatment for hypertension elsewhere?

a. Yes
b. No

If yes, proceed to 19. and 20.
If no, skip to 21.

19. If yes, where were you receiving hypertension treatment previously?

a. Other government facility (e.g. Community clinic, Upazila health complex, Union health complex, District hospital)
b. Medical college hospital
c. Local pharmacy
d. Private physician or private hospital
e. Alternative medicine (Homeopathic/ Ayurvedic/Unani)
f. Other, please specify _________________

20. If yes, Do you spend less money each month out-of-pocket, that is paying your own money, for medical services or medications for hypertension since you started coming to the UHC for hypertension treatment compared to how much you spent before?

a. Yes
b. No

21. In the past 12 months, were there times when you had a serious problem paying or were unable to pay your medical bills?
    a. Yes
    b. No
22. In the past 2 months, did flooding prevent you from visiting the UHC for a blood pressure check?
    a. Yes
    b. No
23. In the past 2 months, did flooding prevent you from getting a refill of your blood pressure medication(s)?

a. Yes
b. No
